# Clinical signs associated with earlier diagnosis of children with autism Spectrum disorder

**DOI:** 10.1186/s12887-021-02551-0

**Published:** 2021-02-25

**Authors:** Nachum Sicherman, Jimmy Charite, Gil Eyal, Magdalena Janecka, George Loewenstein, Kiely Law, Paul H. Lipkin, Alison R. Marvin, Joseph D. Buxbaum

**Affiliations:** 1 Columbia University, Graduate School of Business, 511 Uris Hall, 3022 Broadway, New York, NY 10027 USA; 2 Department of Sociology, Columbia University, New York, NY USA; 3 Seaver Autism Center for Research and Treatment, Icahn School of Medicine at Mount Sinai, New York, USA; 4 Social and Decision Sciences, Carnegie Mellon University, Pittsburgh, PA USA; 5 Kennedy Krieger Institute and Johns Hopkins School of Medicine, Baltimore, MD USA; 6 Kennedy Krieger Institute and Johns Hopkins Bloomberg School of Public Health, Baltimore, MD USA; 7 Seaver Autism Center for Research and Treatment, Department of Psychiatry, Friedman Brain Institute, Mindich Institute for Child Health and Development, Icahn School of Medicine at Mount Sinai, New York, NY USA

**Keywords:** Autism spectrum disorder, Clinical signs, Symptoms, Early diagnosis, Diagnosis age, Regression trees

## Abstract

**Background:**

The objective of this study is to gain new insights into the relationship between clinical signs and age at diagnosis.

**Method:**

We utilize a new, large, online survey of 1743 parents of children diagnosed with ASD, and use multiple statistical approaches. These include regression analysis, factor analysis, and machine learning (regression tree).

**Results:**

We find that clinical signs that most strongly predict early diagnosis are not necessarily specific to autism, but rather those that initiate the process that eventually leads to an ASD diagnosis. Given the high correlations between symptoms, only a few signs are found to be important in predicting early diagnosis. For several clinical signs we find that their presence and intensity are positively correlated with delayed diagnosis (e.g., tantrums and aggression). Even though our data are drawn from parents’ retrospective accounts, we provide evidence that parental recall bias and/or hindsight bias did not play a significant role in shaping our results.

**Conclusion:**

In the subset of children without early deficits in communication, diagnosis is delayed, and this might be improved if more attention will be given to clinical signs that are not necessarily considered as ASD symptoms. Our findings also suggest that careful attention should be paid to children showing excessive tantrums or aggression, as these behaviors may interfere with an early ASD diagnoses.

**Supplementary Information:**

The online version contains supplementary material available at 10.1186/s12887-021-02551-0.

## Background

Given evidence that the timing of interventions can have a large impact on trajectories of children diagnosed with autism spectrum disorder (ASD) [1–7], timing of diagnosis has assumed increasing significance. Timely diagnosis not only enables early clinical and educational intervention, but also relieves parental distress [8, 9], which in turn ameliorates secondary impacts of ASD [10]. Obtaining an accurate estimate of the number of children affected across different age categories is also essential to guide policies and plan service needs [11].

There is extensive evidence, from both population and clinical samples, that ASD can be reliably diagnosed before the age of 2 [12–17], yet the Center for Disease Control and Prevention (CDC) estimates that the median age at diagnosis is 3 years 10 months for more severe autistic disorders, 4 years 1 month for pervasive developmental disorder – not otherwise specified, and 6 years 2 months for Asperger disorder [12]. Thus, most children who are ultimately diagnosed with ASD are not diagnosed until after the age of 4, despite the fact that parents often express concerns a year or two before this age [17]. Family members and caretakers are typically the first to raise concerns, usually before the second year of age [13, 15, 18].

Numerous studies show that both the prevalence and age of diagnosis are correlated with family socioeconomic status (SES). Children of more educated and wealthier parents are more likely to be diagnosed with ASD, and to be diagnosed earlier. With regard to race and ethnicity, the evidence is mixed. While minority children are less likely to be diagnosed with ASD [19], differences in age of diagnosis across racial and ethnic groups are not significant after controlling for parents’ wealth and education [11, 19–21]. Other factors shown to be correlated with age of diagnosis are family structure (e.g., the presence of grandparents in the household) [22], birth order, the type of diagnosis (e.g., Asperger being diagnosed later than other conditions), and level of urbanization; children in urban areas are diagnosed significantly earlier [23].

In the US, the median age at diagnosis is highly variable across states [12, 21]. Rates of diagnosis in different states are affected by the availability of resources [23, 24], distribution of clinics, density of doctors, existence of early screening and intervention programs, and strength of parents' organizations. Rates of diagnosis also correlate with the type of welfare regime in the state, and the state’s history of de-institutionalization for intellectual disabilities [25]. Across states, however, the age of diagnosis is decreasing over time [12, 23], suggesting continuous improvements in ASD detection.

Given a lack of clinical biomarkers for ASD, currently, diagnosis typically relies on both behavioral observation by a qualified clinician and parental report of developmental history and current presentation. Clinical signs are clearly important, given that family members, caretakers, and health-care professionals, who are typically the first to raise concerns, necessarily rely on observations of signs to initiate the processes that eventually lead to diagnosis and treatment. However, only a small number of studies have examined the correlation between the age of diagnosis and the presence and severity of various specific signs, either separately or jointly [26, 27]. Several studies have shown that *overall* severity leads to earlier diagnosis [11], and that specific or broad categories of signs are associated with either earlier or later diagnosis [28]. However, we are not aware of any study that systematically looks at a large set of symptoms and signs and their level of severity, their correlation with one-another, and their connection to age of diagnosis.

The objective of the research reported in this article is to disentangle the relationship between symptoms, clinical signs, and age of diagnosis, utilizing a new, large, online survey of parents of children diagnosed with ASD. Our goal is to statistically identify the clinical signs that are most diagnostic of an early age of diagnosis.

## Methods

### Sample

An online survey, approved by the Columbia University Institutional Review Board (IRB), was completed by 1,815 parents of children who were previously diagnosed with ASD. Potential participants, who were contacted by email, were selected from the Interactive Autism Network (IAN) Research Database and Registry at the Kennedy Krieger Institute, Baltimore, MD.

The IAN Research database and research registry was designed to facilitate ASD research efforts by informing participants about studies for which they qualify. All the participants in the database are parents of children whose ASD diagnosis has been clinically validated [29, 30] as well as verified by a review of parent- and professional-provided medical records [31]. To date, IAN Research has provided recruitment and/or data services for more than 500 research studies. IAN Research is governed by a Johns Hopkins Medicine IRB (NA_00002750; PI: Dr. Paul H. Lipkin).

It is difficult to estimate response rate. We estimate that the recruitment email went to approximately 11,300 e-mail addresses (excluding emails of participants reported as deceased, bounced emails, and emails excluded per participant request). However, we cannot tell how many emails were actually received and read. Thus, the response rate is at least 16%.

Demographic and socio-economic information on parents collected in the study included ethnicity, education, household income, and urban/rural residence. Data from the survey was supplemented with information previously collected by IAN on each of the children in our sample.

In cases where more than one child in the family was diagnosed, parents were asked to answer the survey with regard to their first diagnosed child. After deleting cases for which crucial variables were missing (e.g., age of diagnosis), the sample size was reduced to 1743. Descriptive statistics of the sample as well as other variables that were used in some of the analyses are reported in Additional file 1: Appendix Table 1 (Supplemental Material). These variables are also listed in the “Covariates” section below.

### Survey

This survey is part of a larger project that addresses various factors that could affect the age of diagnosis. In this study we focus on a limited number of questions from the survey. Parents were provided with a list of 25 signs and asked to report those exhibited by their child around the time of his/her diagnosis. Most signs were taken from the diagnostic criteria for autism from the Diagnostic and Statistical Manual for Mental Disorders (DSM). Some modifications to the list were made following a pilot study in which parents also listed signs that are not unique to individuals with autism [22]. This prior study found that there were some signs that, while not unique to ASD, were frequently cited by parents, and may have led them to seek professional help and to obtain a diagnosis.

For each clinical sign or symptom, the respondent could indicate, using a slider, the level of severity ranging from “none” to “severe.” Other intermediate levels were labeled (at equal intervals) as “minor,” “moderate,” and “serious.” Respondents could position the slider anywhere along the scale. Responses were translated to a numerical value on a 0–10 scale. A zero response indicates that a child did not display that particular symptom. The list of the symptoms that parents were asked to assess is presented in Table 1.

**Table 1:** Reported Clinical Signs & Symptoms and Level of Severity

Clinical Signs & Symptom	Obs.	Level of Severity on a 0–10 scale			
		Mean	Median	Std.	% Non Zero
**Social and Communication**					
Delayed speech	1609	6.18	7.40	3.58	0.83
Delayed response to name	1609	4.66	4.90	3.51	0.77
Poor eye contact	1609	6.07	6.30	2.99	0.91
Lack of gestures (e.g., pointing, nodding or shaking head)	1609	5.23	5.30	3.54	0.82
Difficulty understanding gestures	1609	5.08	5.20	3.38	0.83
Preference to play alone or play with objects rather than with others	1609	7.10	7.80	2.90	0.93
More focus on objects than people	1609	6.79	7.50	2.96	0.92
Failure to initiate or respond to social interactions	1609	6.94	7.70	2.80	0.94
Difficulties in initiating and/or maintaining relationships and friendships	1609	7.48	8.30	2.81	0.94
**Restricted and Repetitive behaviors**					
Played with toys or objects in an unusual way (e.g. repetitive play, lining up toys)	1609	6.19	6.60	3.02	0.92
Need for sameness (e.g. difficulties with changes in routine)	1330	6.30	6.90	3.01	0.76
Unusual motor mannerisms (e.g. hand flapping, spinning)	1330	5.29	5.40	3.41	0.71
Unusual interest in specific objects or toys (e.g. high in intensity or focus)	1330	6.45	7.10	3.00	0.75
**Sensory Symptoms**					
Unusual interest in sensory aspects of the environment (e.g. **excessive smelling** of objects or people, **fascination** with lights or movement)	1609	4.77	4.90	3.16	0.86
Sensory hyperreactivity (e.g. excessive or adverse response to specific sounds, lights, touch, smell or tastes)	1609	6.16	6.50	3.01	0.93
Sensory hyporeactivity (e.g. insensitivity or indifference to sensory pain or temperature, slow response to sensory stimuli in the environment)	1609	4.81	5.00	3.39	0.83
**Aggression**					
Temper tantrums	1609	4.85	5.00	3.20	0.85
Aggression toward self	1609	2.48	1.30	3.01	0.60
Aggression toward others	1609	2.65	1.80	3.01	0.64
**Regression** (At some point in time, did x’s behavior regress (deteriorate) in any of the following ways?)					
Loss of skills	1609	2.31	0.10	3.12	0.50
Loss of language (words only)	1609	2.68	0.10	3.60	0.49
Loss of language (phrases)	1609	2.16	0.00	3.46	0.41
Less social engagement	1609	2.96	1.20	3.49	0.56
Loss of motor skills	1609	1.18	0.00	2.36	0.35
Loss of daily living skills	1609	1.56	0.00	2.80	0.38

The smaller number of observations for three of the clinical signs was the result of a technical problem that resulted in a loss of data

### Covariates

In addition to the detailed description of clinical signs, we control in some statistical analyses for socio-economic variables such as ethnicity, parents’ education, family income, and location (urban vs. rural). Other covariates that we include in some analyses are year of birth, to account for the overall increase in diagnosis over time, and a dummy variable for a diagnosis of Asperger, to account for the fact that such children tend to be diagnosed later. Given the retrospective nature of the study, we also use the time elapsed between the time of diagnosis and the date of survey to check for potential biases (see the Study Limitations section and App. B).

### Statistical analyses

To explore characteristics of the data we examined the median and the distribution of all items on the clinical signs list, as well as the correlation structure between individual signs.

Subsequently, we used two statistical methods to study the link between symptoms and age of diagnosis: factor analysis and regression trees. Each of these methods takes a different approach to addressing the same problem: identifying the patterns of signs that lead, most reliably, to early diagnosis of ASD.

#### Factor analysis

Factor analysis is designed to distill multiple variables into smaller sets of factors representing key sources of unique – i.e., non-redundant – variance. For example, a large number of questions designed to measure personality could be distilled, using factor analysis, into measures of a much smaller number of specific traits, such as introversion and impulsivity. This approach allowed us to avoid the problems associated with estimating the effects of multiple signs that are highly correlated by replacing them with the scores of a limited number of uncorrelated factors.

#### Regression trees

Regression trees is a class of machine learning models that make it possible to estimate non-linear relationships in an easily interpretable fashion. The estimation procedure is similar to running numerous Ordinary Least Squares (OLS) regressions for each sign, where the dependent variable is the age of diagnosis and the independent variable is a dummy variable indicating high vs. low severity of the sign. In each regression we use a different level of severity to construct the cutoff for the high/low dummy variable. Clinical signs whose severity allows for best prediction of early vs late diagnosis were identified in an iterative and hierarchical way. The regression tree analysis first identifies, for each sign, the level of severity that best splits the sample between children who are diagnosed earlier and those diagnosed later. Then, among all signs, the signs that allows for the best separation of the sample into children with early versus late diagnosis is selected as the first node in the tree. Two branches are then created, one for children with early diagnosis and one for children with late diagnosis. The above procedure is repeated for each branch, creating two new nodes. This process continues at each node of the tree, until no symptom can further split the sample into two groups such that the differences in age of diagnosis between the groups (early and late diagnosis) are statistically different. In the Results section, we provide more details on the tree construction.

## Results

### Sample demographic and symptom characteristics

Additional file 1: Appendix Table 1 reports the socio-economic characteristics of the sample and compares them to that of the US population. Similar to other studies, the children in our sample are from wealthier, more educated, and more urban families than the US average. Representation of minority groups, especially Hispanic and Black, is also lower than their share of the US population. The effects of various socioeconomic variables on the age of diagnosis in our sample are reported in Additional file 1: Appendix Table 2.

The percent of children that, at time of diagnosis, displayed each of the signs listed in the survey, as well as (for those who reported them) their median values of severity, are presented in Table 1. Additional file 1: Appendix Fig. 1 depicts the value distributions of each sign where it is shown that the modal responses for most signs are either zero (indicating that a child did not exhibit the symptom) or the highest severity level [10]. However, there are sufficient numbers of intermediate severity values reported to render severity level a potentially useful input into statistical analyses.

### Correlations between severity and age of diagnosis (Univariate analysis)

**Fig. 1:**
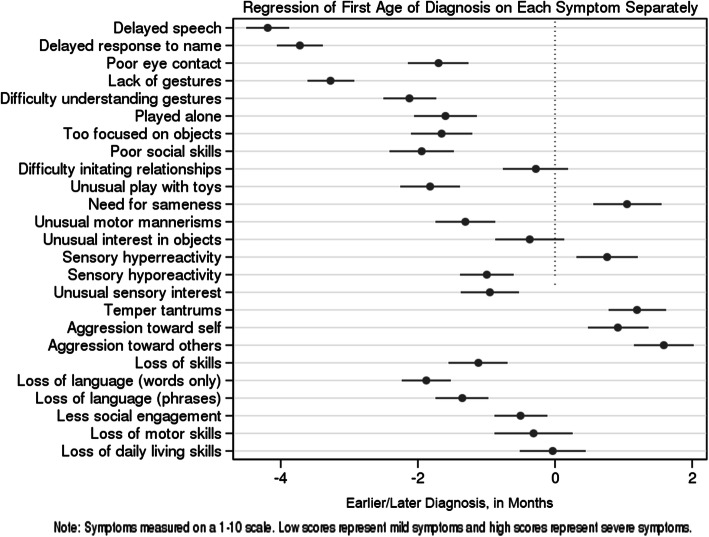
“Correlation Between Clinical Signs & Symptoms Severity and Age of Diagnosis” Note: “Negative” months indicate earlier diagnosis”

Before presenting results using factor analysis and regression trees, we report some basic correlations between each sign and the age of diagnosis. Figure 1 displays graphically, for each sign, the average effect of a one unit increase in reported severity on the age of diagnosis (in months). For example, the number − 2 indicates a decrease of 2 months in the age of diagnosis. Each horizontal line describes the 95% confidence interval for the effect, and the dot in the center displays the point estimate (see Additional file 1: Appendix A for more details).

For most signs, higher severity was predictive of an earlier age of diagnosis (the lines are on the left side of the panel, indicating a negative relationship between severity and age of diagnosis).

Delayed speech, delay in response to own name, and lack of gesture had the strongest effects. Most regressions of skills were associated with an earlier age of diagnosis, except for loss of motor and daily living skills, whose effects were non-significant. However, given that regressive symptoms were less frequently reported by parents, their usefulness for early diagnosis might be limited.

Interestingly, signs associated with aggression as well as “need for sameness” and sensory hyperreactivity are *positively* correlated with the age of diagnosis; children exhibiting these symptoms are diagnosed later, on average.

A limitation of the univariate analysis is that the existence of some signs may be correlated with the existence of others, so that individual signs provide redundant clues about a child’s condition. We use two methodologies to estimate the joint effects of various symptoms on the age of diagnosis: factor analysis and regression trees.

A seemingly natural approach to dealing with this problem would be to estimate the effect of one sign controlling for the effect of each of the others in a multiple regression. This approach proved infeasible due to multicolinearity. The high correlations between many of the signs (see Table 2) increases the variance of the coefficient estimates and makes the coefficient estimates unstable and difficult to interpret. We, therefore, use factor analysis and regression trees to avoid such problems. The estimation results using a multiple regression are reported in Additional file 1: Appendix A.

**Table 2:**
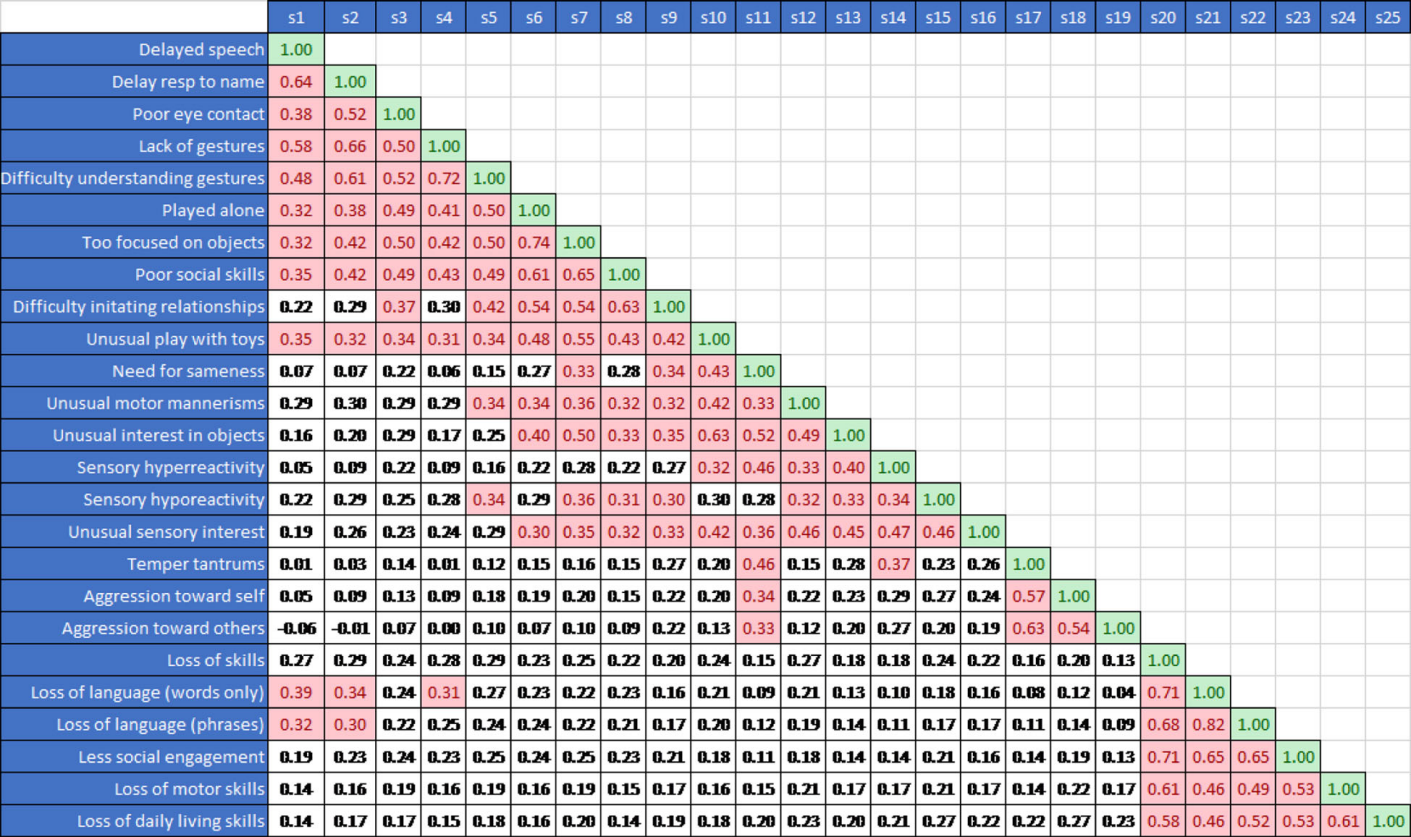
Pairwise Correlations Between Clinical Signs & Symptoms (those highlighted in red are significant at the .05 level)

#### Factor analysis

Factor analysis is most suitable when the correlation between variables of interest is relatively high. A visual examination of the correlations across signs (Table 2) shows a relatively large number of correlations with values of 0.3 and higher (35.7% of the pairwise correlations). Using the Kaiser-Meyer-Olkin (KMO) measure of sampling adequacy, the standard test of whether a data set is appropriate for factor analysis, yielded a value of 0.91, suggesting a high degree of suitability for factor analysis [32].

Using varimax rotation, we identified 5 distinct factors. Table 3 reports the mapping of the 25 signs to these five factors and the labels we chose for these factors. Three of those factors mapped well onto a triad of ASD diagnostic impairments (social interaction, communication, and restrictive/repetitive behaviors). Two additional factors represented items relevant to developmental regression and aggressive behaviors.

**Table 3:** Factor Loadings, 5 Factors, Orthogonal Varimax Rotation, loading>.3

Variable		Factor 1	Factor 2	Factor 3	Factor 4	Factor 5
		Regressive Autism	Social Awkwardness	Communication difficulties	Sensory reactivity and need for sameness	Aggressive Behavior
1	Delayed speech			0.6610		
2	Delayed response to name			0.7492		
3	Poor eye contact		0.4324	0.4654		
4	Lack of gestures			0.7728		
5	Difficulty understanding gestures		0.3613	0.6916		
6	Played alone		0.7254			
7	Too focused on objects		0.7400			
8	Poor social skills		0.6906	0.3184		
9	Difficulty initating relationships		0.6150			
10	Unusual play with toys		0.4290		0.3545	
11	Need for sameness				0.4949	0.3918
12	Unusual motor mannerisms				0.5210	
13	Unusual interest in objects				0.6638	
14	Sensory hyperreactivity				0.4932	0.3374
15	Sensory hyporeactivity				0.3659	
16	Unusual sensory interest				0.5741	
17	Temper tantrums					0.7523
18	Aggression toward self					0.6514
19	Aggression toward others					0.7224
20	Loss of skills	0.8197				
21	Loss of language (words only)	0.8146				
22	Loss of language (phrases)	0.8222				
23	Less social engagement	0.7676				
24	Loss of motor skills	0.6562				
25	Loss of daily living skills	0.6496				

See Table 1 for the full labels of symptoms

The next step in our analysis was to use the weights from the factor analysis to generate factor scores for each child and each factor. The factor scores are then included as independent variables in a regression model in which, as before, the dependent variable is age of diagnosis. Therefore, instead of including all 25 symptoms in one regression, we use the 5 factor scores. This avoids the problem of multicollinearity, since factors are, by construction, uncorrelated with one-another, and, by reducing the number of independent variables, increases statistical power.

The results of this regression are reported in Table 4, Column 1. The factor representing communication difficulties was the strongest predictor of age of diagnosis, with higher levels of severity associated with significantly lower age of diagnosis. Unfortunately, one of the drawbacks of factor analysis is that there is no interpretation to the values of the estimated coefficients.

**Table 4:** Regression Models for the effects of factor scores on age of diagnosis using 5 factor Varimax Rotation Model

	(1)	(2)	(3)	(4)	(5)
Factor 1: Regressive Autism	−2.43***	−2.01***		−1.77**	−1.64**
	(0.66)	(0.65)		(0.79)	(0.78)
Factor 2: Social Awkwardness	0.53	−0.90		0.88	−0.69
	(0.73)	(0.72)		(0.76)	(0.76)
Factor 3: Communication Difficulties	−15.7***	−13.5***		−15.3***	− 13.2***
	(0.70)	(0.74)		(0.77)	(0.79)
Factor 4: Sensory Reactivity & Need for Sameness	−1.97***	−2.55***		−1.61**	−2.34***
	(0.76)	(0.74)		(0.80)	(0.77)
Factor 5: Aggressive Behavior	7.46***	6.25***		7.81***	6.46***
	(0.73)	−0.73		(0.76)	(0.76)
Both Parents Graduated College		−4.04**			−4.07**
		(1.60)			−1.6
Only Mother Graduated College		0.52			0.48
		(1.78)			(1.78)
Only Father Graduated College		−3.65			−3.61
		(2.45)			(2.45)
Black		1.49			1.42
		(2.97)			(2.97)
Hispanic/Latino		3.20			3.24
		(1.97)			(1.97)
Other		3.02			3.06
		(3.46)			(3.46)
Family Income > $100,000		−1.45			−1.48
		(1.54)			(1.54)
Lived in Urban Area		−2.76			−2.76
		(1.79)			(1.79)
Asperger Diagnosis		10.9***			10.8***
		(1.84)			(1.85)
Year of Birth		−0.75***			−0.75***
		(0.10)			(0.10)
Number of Symptoms Reported			−0.97***	−0.31	−0.18
			(0.14)	(0.21)	(0.21)
Constant	46.7***	1549***	64.3***	52.7***	1553***
	(0.62)	(202)	(2.63)	(4.12)	(202)
Observations	1359	1330	1669	1359	1330
R-squared	0.327	0.387	0.030	0.328	0.387

Each column reports the estimated coefficients of one regression

Standard errors in parentheses

*** *p* < 0.01, ** *p* < 0.05, * *p* < 0.1

Developmental regression and restricted-and-repetitive-behaviors (RRBs) were also predictive of earlier age at diagnosis, although much less predictive than communication difficulties. Presence of aggressive behaviors, on the other hand, was associated with a delayed diagnosis. Adding to the regression socio-economic indicators, the child’s year of birth, and an indicator for an Asperger diagnosis (Table 4, Column 2), did not much affect the results.

To test the hypothesis that individual signs play an important role in predicting age of diagnosis beyond what is captured by the overall severity of the child condition, we constructed a measure of overall severity by counting the number of signs that the parents reported with a positive level of severity. The results, reported in Table 4, column 3, show that, on average, a one unit increase in the number of signs reduced the age of diagnosis by almost one month. However, when the regression also includes the five factors representing the effects of the individual signs (Table 4, columns 4 and 5), the effect of overall severity becomes insignificant.

#### Regression trees

We conducted a regression tree analysis using a package called RPART [33]. As described earlier, the first step in constructing the tree is to find, for each sign, the level of severity (on the 0–10 scale) that best split the sample into two groups, those who are diagnosed earlier and those who are diagnosed later. The best cutoff point is produced by the RPART package using “Gini Index” and is, intuitively, similar to choosing a cutoff point that produces the highest explained variation (R^2^) for each sign.

The results of this first step are presented in Table 5. For example, for “delayed speech” the level of severity of 5.75 best divides the sample by age of diagnosis. For children with severity levels of 5.75 or below, the mean age of diagnosis is 63.7 months (median = 58), while for children with levels of severity above 5.75 the mean age of diagnosis is 34.9 (median = 30). The extremely low *p*-values indicate that, with almost certainty, the population mean age of diagnosis for children above the split is different (and lower) than those with severity level below the split.

**Table 5:** Optimal Splits and Mean/Median Age at First Diagnosis Above/Below Split

Clinical Signs & Symptoms	Split	Mean Above	Mean Below	p-value*	Median Above	Median Below
Delayed speech	5.75	34.9	63.7	0.00000	30	58
Delayed response to own name	3.85	35.6	60.4	0.00000	32	52
Poor eye contact	6.15	40.5	52.1	0.00000	33	42
Lack of gestures	6.05	33.6	56.7	0.00000	30	48
Difficulty understanding gestures	3.75	41.1	54.9	0.00000	34	47
Preference to play alone	7.55	42.3	50.7	0.00000	36	40
Too focused on objects	1.05	44.8	61.6	0.00000	36	54
Poor social skills	5.25	43.2	53.9	0.00000	36	42
Unusual play with toys or objects	5.55	41.3	52.7	0.00000	35	42
Need for sameness	2.35	47.4	35.1	0.00000	38	31
Unusual motor mannerisms	3.15	42.6	52.7	0.00000	35	40
Unusual sensory interest	1.65	47.3	35.2	0.00000	38	28
Sensory hyperreactivity	8.55	39.4	47.3	0.00019	33	38
Sensory hyporeactivity	1.75	44.5	51.2	0.00042	36	39
Temper tantrums	2.35	48.2	39.6	0.00000	38	32
Aggression toward self	2.65	51.4	43.3	0.00000	40	36
Aggression toward others	2.15	50.8	41.9	0.00000	41	34
Loss of skills	5.15	39.8	47.5	0.00011	35	38
Loss of language (words only)	5.8	34.8	49.3	0.00000	30	39
Loss of language (phrases)	7.25	35.7	48.0	0.00000	32	38
Difficulty initating on maintaing relationships	“n.a.”	“n.a.”	“n.a.”	“n.a.”	“n.a.”	“n.a.”
Unusual interest in objects or toys	“n.a.”	“n.a.”	“n.a.”	“n.a.”	“n.a.”	“n.a.”
Less social engagement	“n.a.”	“n.a.”	“n.a.”	“n.a.”	“n.a.”	“n.a.”
Loss of motor skills	“n.a.”	“n.a.”	“n.a.”	“n.a.”	“n.a.”	“n.a.”
Loss of daily living skills	“n.a.”	“n.a.”	“n.a.”	“n.a.”	“n.a.”	“n.a.”

* The *p*-value is for the difference between the means

At the bottom of the table are signs for which no level of severity could separate the sample into two groups where the age of diagnosis of one group was significantly different from that of the other.

**Fig. 2:**
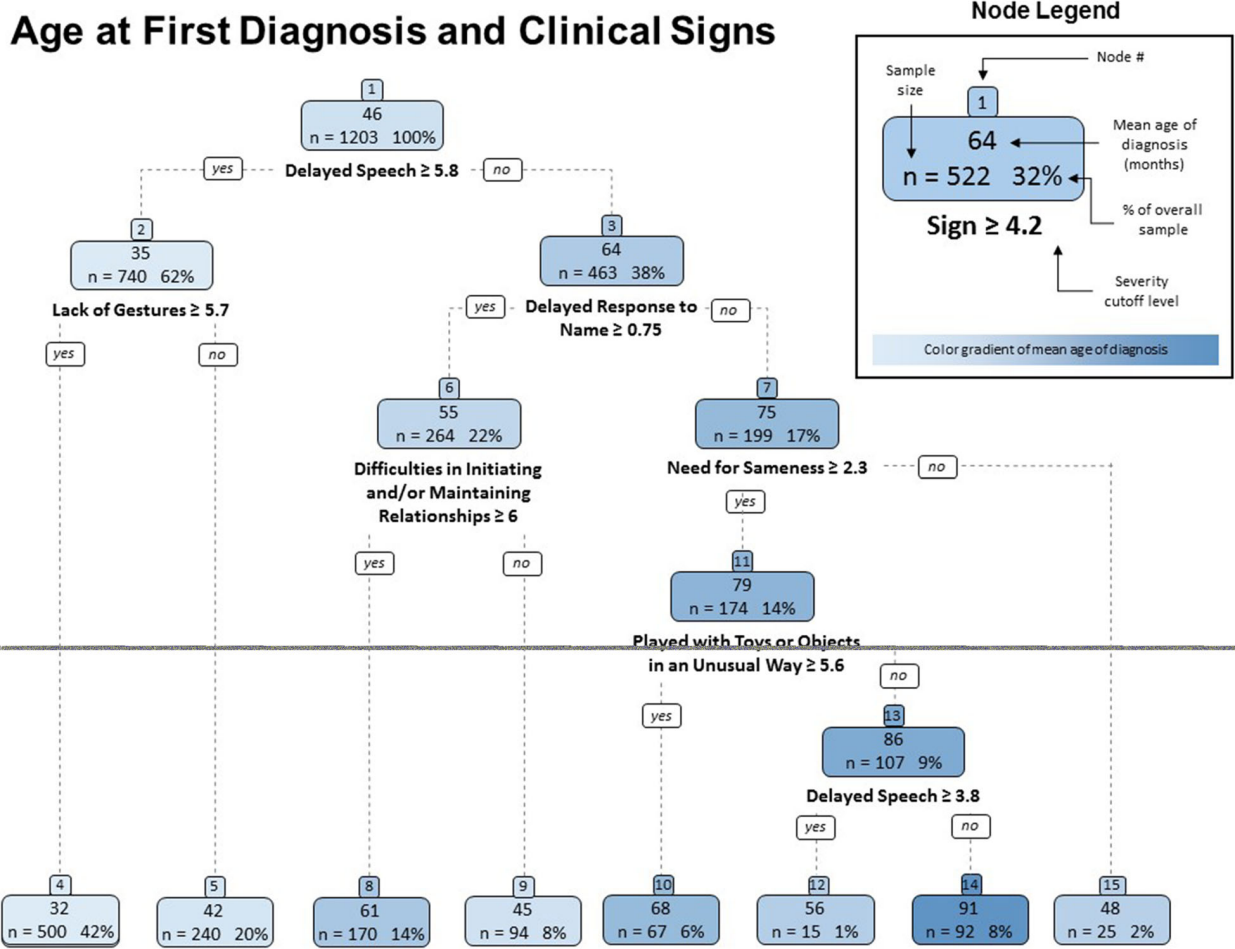
“Regression Tree of Age of Diagnosis and Clinical Signs & Symptoms”. Note: Out of the sample of 1743, only 1330 respondents were able to see the full list of clinical signs and symptoms (see footnote to Table 1). Only 1203 of the 1330 also had a valid age of diagnosis reported

In creating the tree (Fig. 2) we work from top down, first picking the sign for the top of the tree that best divides the sample between children who are diagnosed earlier and those who are diagnosed later, based on the criteria discussed above. This is “delayed speech,” which, with a cutoff level of 5.8 (all cutoffs numbers are rounded in the figure) splits our sample into two groups: 62% (*n* = 740) with a severity level of 5.8 or above have an average age of diagnosis of 35 months, and 38% of the sample (*n* = 463), with a severity level below 5.8, have an average age of diagnosis of 64 months.

Next, for each of these sub-groups, we again split the sample, using the same criteria. We repeat this process until we cannot split the sample into two sub-groups such that the difference in the age of diagnosis is statistically significant.

From Fig. 2, we can see that when we limit the sample to the children with high severity of delayed speech (node 2, where severity level is ≥5.8), the only remaining sign that further splits this sub-sample is “lack of gestures” where the cutoff level of 5.7 splits our sample into two groups (nodes 4 and 5): 42% (*n* = 500) with a severity level of 5.7 or above have an average age of diagnosis of 32 months, and 20% of the sample (*n* = 240), with a severity level below 5.7, have an average age of diagnosis of 42 months. As the graph shows, these two groups, both relatively large, cannot be further divided.

Going back up the tree, of children with relatively low severity of delayed speech (node 3), the symptom that best divides this sample is “delayed response to name.” Notice that here the cutoff level is quite low (0.75), suggesting that among children with no (or low level of) delayed speech, any level of delayed response to name is important in predicting the age of diagnosis. The cutoff level of 0.75 splits the subsample into two groups (nodes 6 and 7): 22% (*n* = 264) with a severity level of 0.75 or above have an average age of diagnosis of 55 months (node 6), and 17% of the sample (*n* = 199), with a severity level below 0.75, have an average age of diagnosis of 75 months (node 7). Again, the rationale for such a split seems intuitive, at least after the fact. Speech could be delayed for a variety of reasons and the delay can take many forms, for example, an ability to receive and understand speech without the capacity to speak. If the child does not respond to his name, however, the lack of response is likely to be more diagnostic, to be indicative of a lack of attachment or lack of social awareness, “autistic aloneness.” Combined with difficulties in initiating relationships we get the “Asperger” type and a later diagnosis (node 8).

Looking at the sample represented by node 6, we see that the sign that best splits this sample is “difficulties in initiating and/or maintaining relationships.” The cutoff level of 6.0 splits our sample into two groups (nodes 8 and 9). Fourteen percent (*n* = 170) with a severity level of 6 or above have an average age of diagnosis of 61 months, and 8% of the sample (*n* = 94), with a severity level below 6, have an average age of diagnosis of 45 months. It should be noted that here, children that exhibit the sign at higher severity are actually diagnosed at an older age.

Moving up to the sample represented by node 7 (low levels of delayed speech and low levels of delayed response to own name), the symptom that best split the sample is “need for sameness,” and the cutoff level of 2.3 splits our sample into two groups (nodes 11 and 15). Here again, children who exhibit the sign at higher severity are diagnosed at an older age. The 14% (*n* = 174) with a severity level of 2.3 or above have an average age of diagnosis of 79 months, and the 2% of the sample (*n* = 25), with a severity level below 2.3, have an average age of diagnosis of 48 months. This group cannot be further split, most likely due to small sample size.

Going back to node 11, the sign that best split this sample is “played with toys or objects in an unusual way.” The cutoff level of 5.6 splits our sample into two groups (nodes 10 and 13). Children with the higher level of severity have a lower age of diagnosis. The 6% of our sample (*n* = 67) with a severity level of 5.6 or greater have an average age of diagnosis of 68 months, and 9% of the sample (*n* = 107), with a severity level below 5.6, have an average age of diagnosis of 86 months. Notice that this sub-group has the highest age of diagnosis so far. Looking at this specific subgroup we see that once again “delayed speech” is the sign that best splits it. Note that we have here a small sub-group of children with relatively low levels of delayed speech. However, when limiting the sample to this sub-group, those with relatively more delayed speech are diagnosed earlier. A cutoff level of 3.8 divides the group into our last two subgroups, represented by nodes 12 and 14. Children with delayed speech levels of 3.8 or above are diagnosed at an average age of 56 months, and children with severity level below 3.8 are diagnosed at an average age of 91 months. The groups’ sizes are 15 (1%) and 92 (8%) respectively.

In sum, the regression tree analysis identifies speech delay, lack of gestures and delayed response to name – all components of factor 3 in the factor analysis - as the key signs leading to early diagnosis (the lighter the node’s color in Fig. 2, the earlier is the diagnosis).

## Discussion

Our study analyzed, using several different analytical approaches, variation in age of diagnosis as a function of a child’s signs and symptoms at time of diagnosis. Leveraging a unique sample of over 1600 children with an ASD diagnosis, for whom detailed demographic and clinical information was available, allowed us to explore a variety of clinical factors associated with age of diagnosis, while controlling for key environmental factors identified by previous studies (SES, urban/rural residence and cohort).

The different methodologies we employed yielded overlapping, but informatively distinct findings. All analyses strongly point towards deficits in early communication as an important factor in early diagnosis, findings that corroborate and build on previous research. We showed that deficits in early communication are predictive of age at diagnosis over and above overall symptom severity. Importantly, these symptoms were observed by parents much earlier than the current median age of diagnosis. This suggests the potential value of designing targeted tools that could enable family members to identify key early symptoms of ASD before they come to the attention of healthcare specialists.

### Deficits in communication as the earliest indicators of ASD

Our results indicated that early deficits in communication – particularly responding to one’s name, lack of gestures, and delayed language, − are all strongly associated with an earlier age at diagnosis. Using a machine learning approach, we show that children whose parents reported the presence of high severity of those signs were diagnosed with ASD 33 months earlier compared to children whose parents either did not observe those sings or observed only their milder presentations.

The finding that early deficits in communication are predictive of early diagnosis is consistent with earlier studies which found that problems with verbal and nonverbal communication, rather than abnormal social interactions or presence of restricted/repetitive behaviors, are key for early detection of ASD [27]. Using, to our knowledge, one of the biggest sample to date, we replicated the finding from a number of studies that parental concerns about child’s communication are the earliest signs of an underlying ASD [34–36]. As we noted earlier, the most likely explanation for this finding is that these symptoms index what parents expect of normal development in the home in the first 2 years of life. If we think of child development not only as a process of biological maturation, but also as a social “career” [25, 37] in the course of which children transition from simpler to more complex social situations, it stands to reason that the factors predictive of early diagnosis would involve the most basic skills of social communication. These signs are likely to trigger a referral to early intervention programs, where such programs exist (recall the finding that early diagnosis is strongly correlated with urban residence). Other signs more strongly indicative of ASD will then become more evident against the background of the type of interactions expected in these programs, in which therapists work one-on-one with children and often involve children in group activities. Given the experience of early intervention workers with many types of disabilities.

Daniels and Mandell (2014) speculated that the later age of diagnosis in Asperger’s Syndrome vs. autism is due to lack of speech delay in the former, making it less evident until children move into the more socially demanding environment of school. Chawarska and colleagues provide evidence for such heterogeneity in the early course of ASD, and propose that the lack of such communication problems in early development may mask social difficulties until later in development, contributing to a delay in the diagnosis [38, 39]. Similarly, several studies suggested that restrictive/repetitive behaviors appear to worsen with age [40–43]. Lord (1995) speculates that, since these behaviors are also present in normally developing young children, the worsening may reflect the fact that “parents become better able to recognize the absence of normal development as children grow older” (page 1377). This improved ability owes, no doubt, to a greater range of experiences and comparisons as children begin to exit the home environment, and as normally developing children respond more appropriately to the demands of new environments. Collectively, this evidence suggests that some of the efforts towards reducing the age at diagnosis should better reflect how the disorder manifests differently over time due to both maturational changes, and the transitions in social environments that children typically undergo. The full set of signs required for a formal diagnosis are not likely to emerge until later in development; yet by this time an important window of opportunity for early intervention may have been missed. This argument is supported by studies showing that clinician judgement, rather than use of standardized diagnostic criteria, was associated with an earlier and more stable diagnosis [44]. It is also in agreement with a tri-level model of autism screening and diagnosis, the first level of which consist of generic developmental surveillance as soon and as often as possible and the second which involves referral to early intervention when necessary. Only at the third level, which would typically follow early intervention at a somewhat later age, would the full and formal diagnostic schedule become relevant.

### Factors associated with a delayed diagnosis

Our results suggest that temper tantrums and aggression as well as “need for sameness” and sensory hyperreactivity are *positively* correlated with the age of diagnosis; children exhibiting these symptoms are diagnosed later, on average.

Note, moreover, that these clinical signs are relatively uncorrelated with all other signs in Table 3 (apart from “need for sameness”). This suggests that this cluster is associated with a particular ASD subtype, the presentation of which is milder, and hence does not become obvious until the child is older and has transitioned into relatively complex and socially demanding settings. Indeed, the diagnostic delay associated with these symptoms may itself be a cause of those symptoms, which emerge as a consequence of non-recognized ASD-related difficulties.

An alternative explanation is that such clinical signs may mask other ASD-associated signs. For example, an aggressive child would be first treated for aggression and not much else and then, over time, additional diagnoses would be considered This in turn indicates that children showing excess temper tantrums and/or aggression should be evaluated for autism, rather than awaiting more obviously ASD-linked signs.

### Study limitations

One limitation of our study is that the sample is limited to children who were eventually diagnosed with ASD, and hence does not include information on the prevalence and severity of clinical signs among children who were not diagnosed with ASD. As a consequence, we could only establish their connection to age at diagnosis, but not their overall usefulness in predicting which children will go on to develop ASD. Collecting such information would, however, be difficult. The relevant population to sample for signs could include all children, children identified as being “at risk,” or children with a demographic profile similar to that of children who are diagnosed with ASD. Use of each of these comparison populations would likely yield different results.

Given the retrospective nature of our study, a potential concern could be that the accuracy and quality of responses could diminish with the time elapsed since the child was diagnosed until the survey date. To deal with this issue, we conducted several statistical analyses to test for such bias (see Addtional file 1: Appendix B). First, we tested, for each sign, whether the probability that it would be recalled by the parent was influenced by the passage of time; it was not. Second, we conducted a similar test, but for the severity of each sign. Again, reported severity levels were unrelated to time passed since diagnosis. Finally, we included time elapsed since diagnosis as a covariate in our regression analyses. Doing so did not affect our findings.

Parents’ recall of the type of clinical signs and symptoms exhibited by their child prior to diagnosis could also be distorted by their awareness, at the time of completing the survey, of their child’s diagnosis. However, our results run contrary to what one would expect if such a bias were present. If parents’ recall was influenced by current diagnosis in a fashion consistent with hindsight bias – the tendency to believe that one knew in the past what one knows in the present – then they would be more likely to emphasize symptoms that are specific to autism, i.e., that differentiate autism from other conditions, yet we find the opposite. We caution, however, that the same does not necessarily hold for parents’ recall of the *severity* of their children’s signs. Consequently, we have less confidence in our results regarding severity.

These limitations arising from potential recall biases occur because of the retrospective nature of the study. Prospective epidemiological research on this question is, however, impractical. Only a small fraction of all children are diagnosed with ASD; hence an epidemiological sample of parents of children who have not yet been diagnosed with ASD will contain too few cases of children who will end up with ASD diagnosis later. For this reason, we believe that despite the well-known problems with retrospective studies, our approach – especially given the direction of our results and the additional tests we conducted to rule out retrospective bias – is the best way to shed light on this crucial question.

Another limitation is that questions about signs refer to only one point in time: around the time of the diagnosis. Clinical signs that appear later and might have raised concerns had the child not already been diagnosed, as well as signs that occurred earlier but did not reach the threshold for parents or other care-givers to delve further or seek help, were not reported, and hence not used in the analysis. In addition, given that the signs and their level of severity are reported by the parents, they are, by definition, subjective and could be biased.

We also cannot infer causation between the sign profile and age of diagnosis, and the direction of many of the effects reported in our study remains to be established. For example, playing alone could *cause* children to be diagnosed later, perhaps because it reduces the salience of other cues, such as delayed speech. However, it is more likely that playing alone is associated with late diagnosis because it becomes apparent only later, when children transition to environments where they are expected to play with other kids.

Even when we suspect that a causal chain does exist, identification of the exact pathway of causation cannot be established. It is likely, for example, that delayed speech contributes to early diagnosis not because parents identify it as a cause for concern about ASD, but because it leads to referral to speech experts, who are more attune to the possibility of ASD as well as familiar with other patterns of signs that predict it.

Finally, similar to most studies of children with ASD, the indices of socioeconomic status were higher in our sample than in the general US population. If this discrepancy results, in part, from a greater willingness to participate in surveys on the part of high SES individuals, then this could affect the generalization of our findings to the broad population. However, the general findings of our study should still hold unless the path to diagnosis is systematically different for lower SES families.

## Conclusion

Utilizing a large survey of parents of children with ASD, this is the first study that systematically looks at a large set of clinical signs and symptoms, their presence and level of severity, the correlations between them, and how they are related to the age of diagnosis. We show that individual signs play an important role in predicting age of diagnosis beyond what is captured by the overall severity of the child condition.

Since many signs are highly correlated, we show that most of the variation in age of diagnosis can be captured by a small number of signs. Using regression tree, we can both rank-order the signs and show how they interact with each other to provide an earlier diagnosis.

While some of our findings are consistent with practitioners’ experience, this study provides statistical support for existing intuitions, as well as new insights. A key new insight is that clinical signs not specifically associated with ASD can lead to earlier diagnosis if they bring the child to the attention of professionals who have greater experience with, and hence greater skill in diagnosing, ASD. Another key insight is that careful attention should be paid to children showing excessive tantrums or aggression, as these behaviors may interfere with an early ASD diagnoses. Similarly, in the subset of children without early deficits in communication, diagnosis is delayed, and this might be improved if more attention will be given to clinical signs that are not necessarily considered as ASD symptoms.

## Supplementary Information

**Additional file 1: Appendix Table 1.** Sample Socio-Economic Characteristics. **Appendix Figure 1.** Distribution of Clinical Signs & Symptoms by Levels of Severity. **Appendix Table 2.** The Effects of Various Variables on Age of Diagnosis. **Appendix A.** Regression Analyses. **Appendix Table A1.** Regressions of First Age of Diagnosis on Symptom Severity. **Appendix Figure A1.** The Effect of all Clinical Signs & Symptoms (Combined) on Age of Diagnosis. **Appendix B:** Testing for potential recall-bias. **Appendix Table B1.** Child age at diagnosis, at time of survey, and time elapsed. **Appendix Table B2.** The effects of Time Elapsed on the Liklihood that a sign is recalled and the reported severity of the symptom.

## Data Availability

The datasets generated and/or analyzed during the current study are not publicly available due to privacy constraints but are available from the corresponding author on reasonable request.

## Abbreviations

ASD: Autism Spectrum Disorder
IAN: Interactive Autism Network
