# Supplementary material for: Clinical signs associated with earlier diagnosis of children with autism Spectrum disorder
Source: BMC Pediatr. 2021 Feb 25;21:96. doi: 10.1186/s12887-021-02551-0 (PMC7905573; doi:10.1186/s12887-021-02551-0)

**Supplementary Materials**

**Appendix Figure 1: Distribution of Clinical Signs & Symptoms by Levels of Severity**


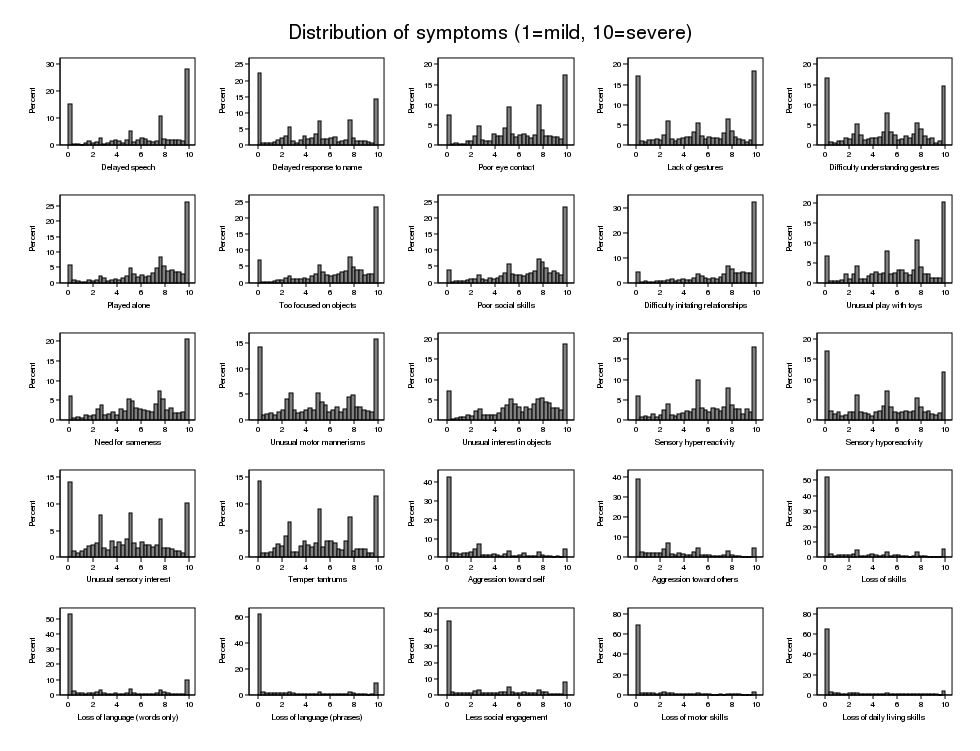

Each column reports the estimation results of one regression. The dependent variable in all 5 regressions is the age of diagnosis (in months). The independent variables vary across regressions and are listed in the first column. For example, parents’ education dummies are included in regressions (1) and (5) only.

**Appendix A: Regression Analyses**

In Appendix table A1 we report the estimation results of various regression analyses where the dependent variable is the age of diagnosis (in months).

Column 1 reports the estimation results of 25 separate regressions. Each cell reports the estimated coefficient and standard errors used to draw the point estimates and confidence intervals in Figure 2 in the paper. Each coefficient measures the average change in the age of diagnosis (in months) for a one-unit change in the severity of the clinical signs and symptom. For example, a one unit increase in the level of severity of delayed speech is associated with, on average, about four months decline in the age of diagnosis.

[PLACE APPENDIX TABLE A1 HERE]

In Appendix Table A1, column 2, we report the estimation result of a multiple regression model. Unlike the first column, here we report the estimated coefficients of a single regression, where the partial effects of all clinical signs and symptoms on the age of diagnosis are estimated simultaneously.

Comparing the results in column 2 to those in column 1, several observations can be made. For many symptoms, while the sign of the coefficient did not change, the effect became weaker and sometimes less significant as it was in the case when it was the only variable in the regression. This is natural, given that multiple regression looks for a symptom’s unique predictive power. In two cases there was a dramatic change in the coefficient. In the univariate regressions, “poor eye contact” and “difficulty understanding gestures” were each negatively (and significantly different from zero) correlated with the age of diagnosis. However, when including all other clinical signs and symptoms in the regressions (i.e., holding other signs symptoms constant), higher severity levels of these two symptoms significantly *increase* the age of diagnosis.

[PLACE APPENDIX FIGURE A1 HERE]

In order to account for other factors that are likely to affect the age of diagnosis, the analyses reported in Appendix Table A1, columns 1 and 2, were repeated by adding the following covariates: parents’ education, race/ethnicity, family income (if over $100,000), residence location (if urban), an Asperger diagnosis, and year of birth.

In Appendix Table 1 we report the effects of these variables on the age of diagnosis, both separately and jointly. Our findings, which are similar to those reported in other studies, show that parents’ education and urban residence, are the two primary facilitators of early diagnosis. They neatly capture the interaction between parent-early intervention and infrastructure that is necessary for early diagnosis. The improvement in diagnosis over time is reflected in the “year of birth” coefficient, showing that, in our sample, for every year that passed, the average age of diagnosis went down by 0.85 months.

Comparing the results in columns 3 and 4 to those in 1 and 2, respectively, show that the inclusion of these additional covariates, while affecting the point estimates, did not change the general findings.

Appendix Figure A1: The Effect of all Clinical Signs & Symptoms (Combined) on Age of Diagnosis


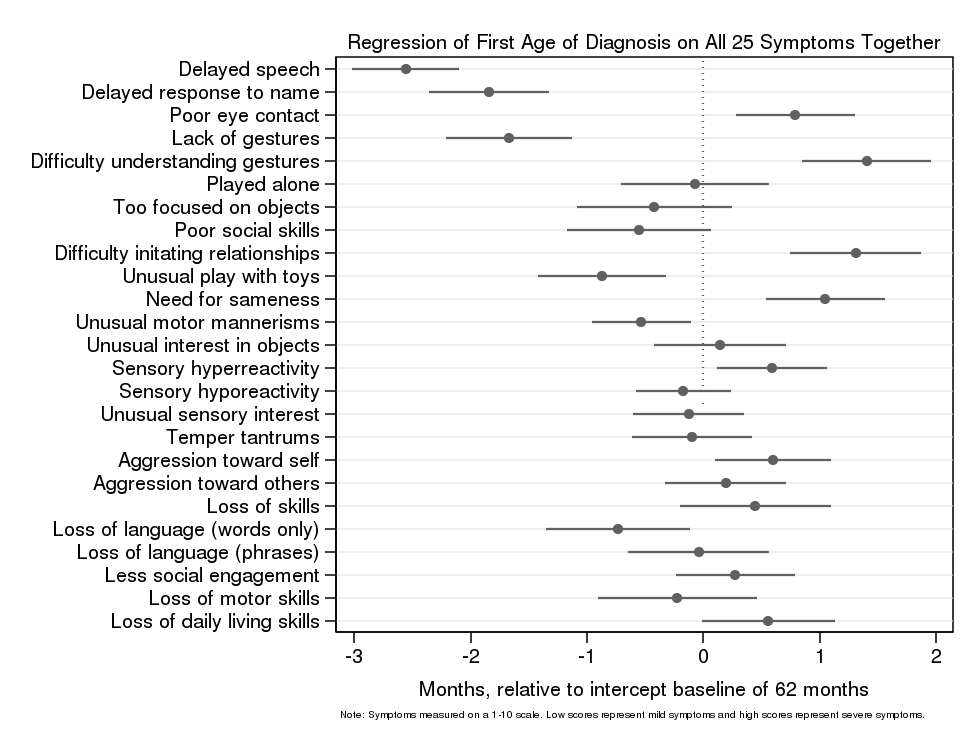


**Appendix B: Testing for potential recall-bias**
Given the retrospective nature of our survey data, it is possible that parents’ ability to recall events that took place when their child was diagnosed will decrease over time. Such a diminishing ability could have two distinct effects: Parents recollection becomes less accurate over time and their recollection becomes biased in a systematic way. In this appendix we test whether time elapsed between the time the child was diagnosed and the survey date, affected parents’ recollections concerning the clinical signs their child exhibited at the time of diagnosis.

Appendix Table B1 provides some descriptive statistics on the distribution values of the age of diagnosis, the child age at the time of survey, and the time elapsed between the two dates. Appendix Figures 1-4 provide visual description of these variables.

In order to test for potential recall bias, we ask the following questions:

1. Does time elapsed since diagnosis affect the likelihood that a clinical sign or a symptom will be reported by parents?
2. Does time elapsed since diagnosis affect the intensity of a sign reported by parents?

In Appendix Table B2 we test these two questions directly. In the first column we report the estimation results of 25 separate regressions. In each regression we estimate the effect of time elapsed on the likelihood that a clinical sign will be reported. For none of the clinical signs, the time elapsed decreases the likelihood that the sign will be recalled by the parents.

In the second column, we estimate, for each clinical sign or symptom, the effect of time elapsed on the level of severity recalled by the parents. For none of the signs the reported severity is decreasing with time. In fact, for many signs and symptoms, the level of reported severity is increasing with time elapsed, although none of the coefficients is significantly different from zero.

To summarize, we find no evidence that time elapsed between the time of diagnosis and the survey has any effect on the two central variables of our analysis, the signs and symptoms reported by the parents and the levels of severity as recalled by the parents.

Our findings are consistent with the idea that as past events are more significant, recall accuracy is higher (Coughlin, 1990). For example, Wilcox and Horney found that the major determinant of spontaneous abortion recall was the length of pregnancy at the time of abortion (Wilcox and Horney, 1984). Only 54% of abortions which accrued within the first 6 weeks of gestation were recalled, compared to 93% of those occurring after 13 weeks.

Appendix Figure B1


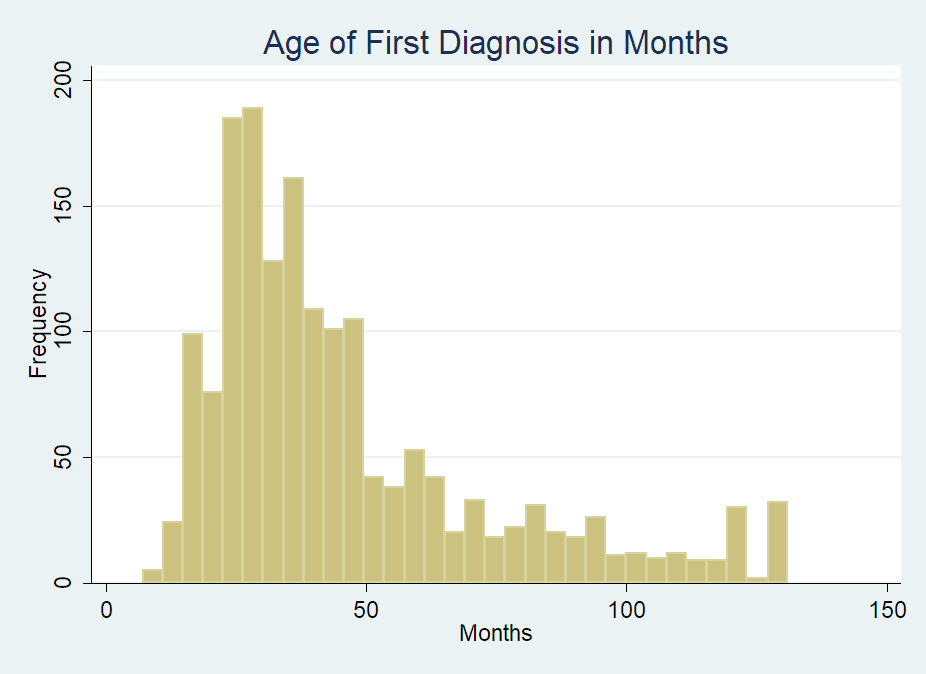


Apnnedix Figure B2


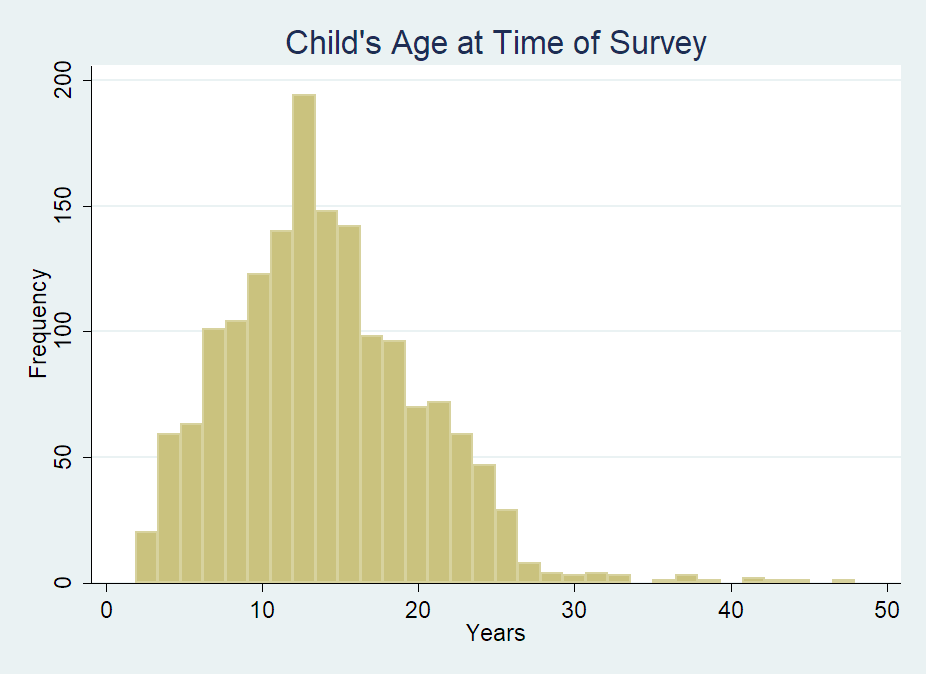


Appendix Figure B3


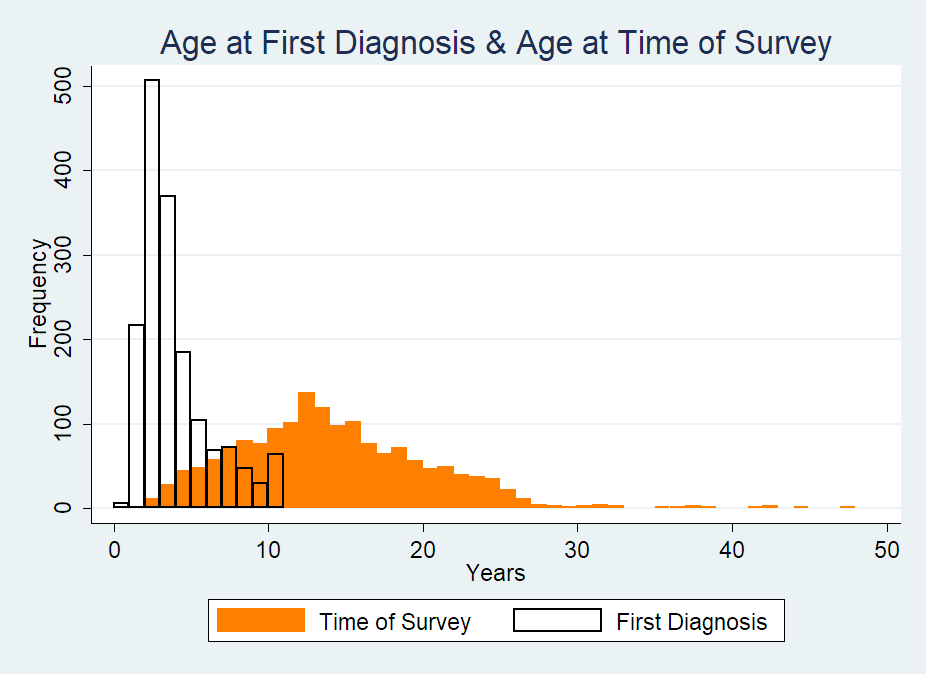


Appendix Figure B4


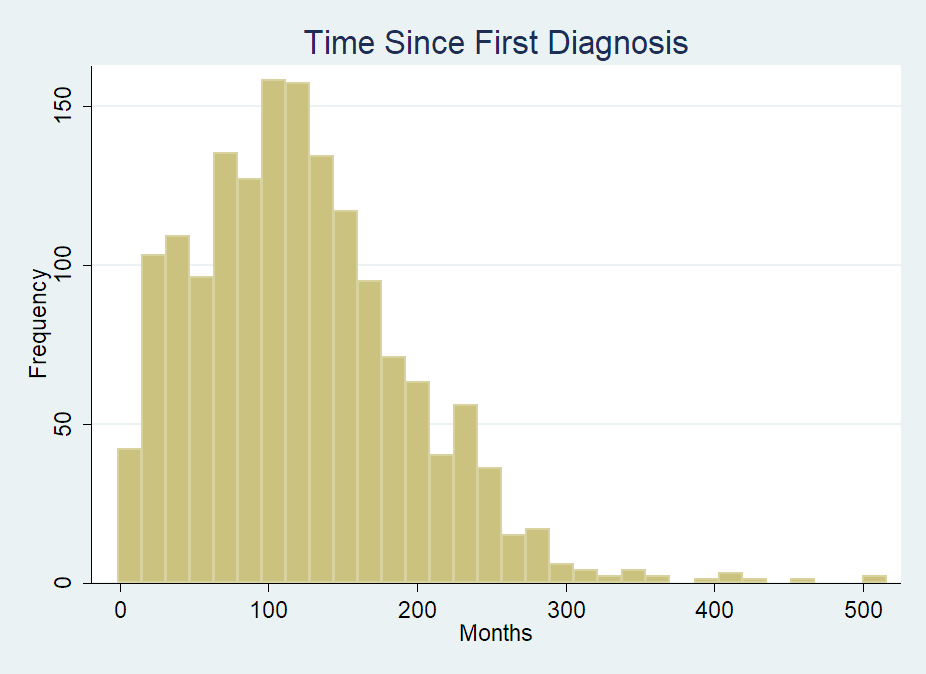

Supplement: Supplementary file 1 — Additional file 1: Appendix Table 1. Sample Socio-Economic Characteristics. Appendix Figure 1. Distribution of Clinical Signs & Symptoms by Levels of Severity. Appendix Table 2. The Effects of Various Variables on Age of Diagnosis. Appendix A. Regression Analyses. Appendix Table A1. Regressions of First Age of Diagnosis on Symptom Severity. Appendix Figure A1. The Effect of all Clinical Signs & Symptoms (Combined) on Age of Diagnosis. Appendix B: Testing for potential recall-bias. Appendix Table B1. Child age at diagnosis, at time of survey, and time elapsed. Appendix Table B2. The effects of Time Elapsed on the Liklihood that a sign is recalled and the reported severity of the symptom. [file 12887_2021_2551_MOESM1_ESM.docx]
